# Study on the Discharge Characteristics of Single-Pulse Discharge in Micro-EDM

**DOI:** 10.3390/mi11010055

**Published:** 2020-01-01

**Authors:** Qingyu Liu, Qinhe Zhang, Min Zhang, Fazhan Yang

**Affiliations:** 1 Key Laboratory of Laser Green Intelligent Manufacturing Technology, Shandong Research Center of Laser Green and High Efficiency Intelligent Manufacturing Engineering Technology, Qingdao University of Technology, Qingdao 266520, China; qingyu-liu@foxmail.com (Q.L.); fazhany@163.com (F.Y.); 2 Key Laboratory of High Efficiency and Clean Mechanical Manufacture of Ministry of Education, School of Mechanical Engineering, Shandong University, Jinan 250061, China; 3 School of Mechanical Engineering, Nanjing Institute of Technology, Nanjing 211100, China; zhangminares@foxmail.com

**Keywords:** micro-EDM, single pulse, discharge characteristic, polarity effect

## Abstract

To further study the discharge characteristics and machining mechanism of micro-electrical discharge machining (micro-EDM), the variation trends of the discharge energy and discharge crater size with actual discharge duration are discussed based on single-pulse experiments. The polarity effect of micro-EDM was analyzed according to the motion characteristics of electrons and ions in the discharge plasma channel. The results show that the discharge current and voltage of micro-EDM were independent of the discharge width and open-circuit voltage. The energy utilization rate of the short-pulse discharge was relatively high, and the energy utilization rate decreased gradually as the discharge duration increased. Even if the mass of the positive ion was much larger than that of the electron, the kinetic energy of the positive ion was still less than that of the electron when bombarding the surface of the electrode. The acceleration and speed of electrons were very high, and the number of times that electrons bombarded the surface of positive electrode was more than 600 times that of positive ions bombarding the surface of the negative electrode during the same time.

## 1. Introduction

With the increasing market demand for miniaturization and the precision of products, micro-processing technology has become one of the important indicators to measure a country’s scientific and technological strength [1,2,3]. Micro-electrical discharge machining (micro-EDM) has become one of the most popular microfabrication technologies due to the advantage of machining electrically conductive materials without direct contact [4,5,6]. Micro-EDM has been has attracted great attention from researchers and has been widely used in the micro-machining field [7,8,9].

Micro-EDM can be seen as a scaled-down version of conventional EDM; however, many influential factors that can be ignored in conventional EDM have a non-negligible influence on micro-EDM performance [10,11,12]. The micro-EDM performance is affected by various conditions, such as processing parameters, and the dielectric and electrode characteristics, which makes it extremely difficult to explain the machining mechanism of micro-EDM. The instability of the discharge state, the extremely high discharge frequency, the very small discharge gap, and the very small discharge energy lead to the large randomness of the shape and size of the discharge crater in micro-EDM [13]. There are two main reasons why it is difficult to study the machining mechanism of micro-scale EDM: one is the randomness and complexity of the discharge process; the other is the lack of sufficient understanding of the oscillation characteristics of the discharge channel, the motion characteristics of charged particles, and the electromagnetic characteristics. In the process of a micro-scale spark discharge, the discharge channel not only expands rapidly, but also exhibits intense oscillation characteristics, and the high frequency oscillation of the discharge channel will have an important impact on the material removal process [14]. Moreover, there is a polarity effect in EDM, that is, the surfaces of the anode and cathode are bombarded by electrons and ions, respectively, during EDM, and they are both corroded by an instantaneous high-temperature heat source. Even if the materials of the two electrodes are the same, the amount removed from the two electrodes is different [15]. By using the polarity effect, the machining performance of micro-EDM can be further improved. However, the machining mechanism of micro-EDM is still unclear and the theory of micro-EDM is still not perfect. The machining performance of micro-EDM is expected to be further improved by studying the theory of micro-EDM, which is lagging. 

Analyzing the characteristics of a single-pulse discharge is the basis for studying the performance of continuous discharge machining, which is a simple and effective method for exploring the material removing mechanism of micro-EDM. Although the process of continuous discharge is much more complicated than that of a single-pulse discharge, the surface roughness and material removal rate can be predicted effectively by studying the characteristics of a single-pulse discharge [16,17]. In this paper, the experimental study of a single-pulse discharge was conducted by taking the discharge duration as an independent variable. The discharge energy and discharge crater size were statistically analyzed, and the variation trend with the discharge duration is discussed in relation to the experimental results. In addition, based on the oscillation characteristics of the discharge channel, the movement of electrons and ions at the discharge point was theoretically analyzed, and the polarity effect of micro-EDM was analyzed and is discussed. 

## 2. Materials and Methods 

### 2.1. Experiment Setup

**Figure 1:**
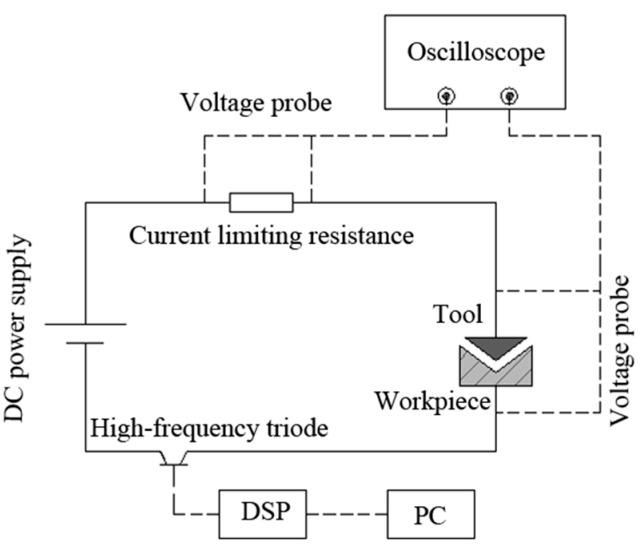
Schematic diagram of a single-pulse discharge system. DSP: digital signal processor.

In this study, a transistor-type power supply system was used to provide energy for a single-pulse discharge. The schematic diagram of the single-pulse discharge system is shown in Figure 1. As shown in Figure 1, the power supply system adopted a direct-current power supply with an adjustable voltage from 0 to 120 V and used a high-speed digital signal processor DSP2407 (Feisheng Electronic Technology Co., Ltd, Shenzhen, China) to send out specific pulses to control the switching on and off of a high-frequency triode, thus generating voltage pulses of a specific pulse width. The minimum pulse width of the power supply was 0.5 μs. 

**Figure 2:**
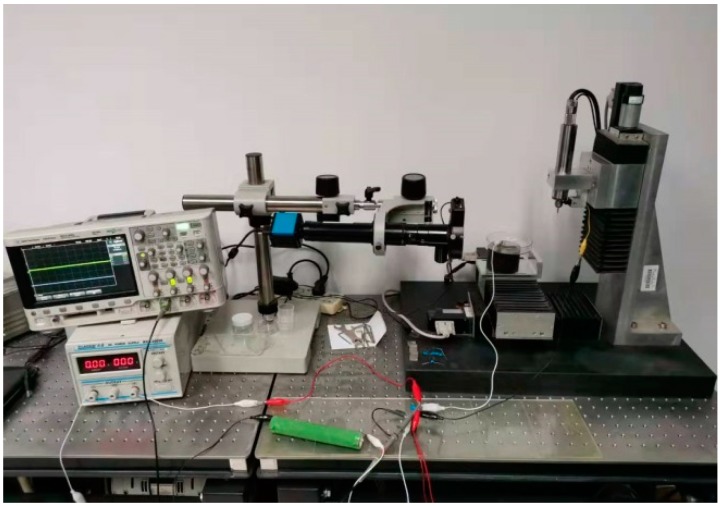
Image of the experimental equipment.

The experiments were conducted on a precision micro-EDM platform. The travel distance of the x-, y- and z-axes was 100 mm, and the motion resolution was 0.2 µm. The solid cylindrical tungsten electrode with a diameter of 200 µm was used as a tool electrode and T2 copper with a thickness of 300 µm was used as a workpiece. The polarity of the tool and workpiece electrodes was interchangeable. An image of the experimental equipment is shown in Figure 2. 

**Figure 3:**
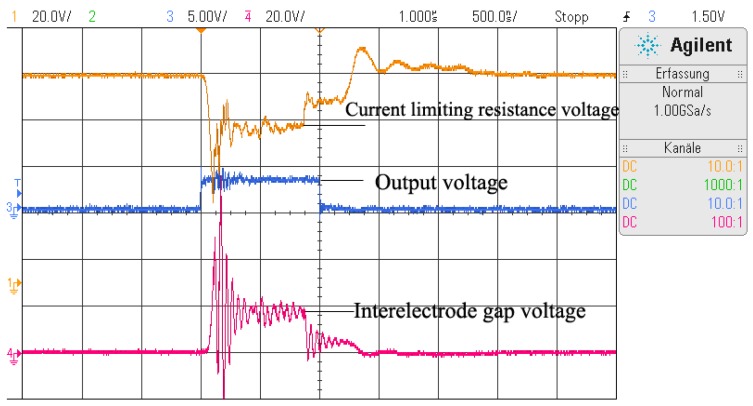
Discharge waveform of a single-pulse discharge.

A current-limiting resistance of 50 Ω was used to limit the discharge current. The open-circuit voltages used in the study were 120 V and 90 V, and the pulse widths were 1 μs, 5 μs, 10 μs, and 20 μs. Each discharge experiment with different discharge parameters were repeated at least 10 times. Because the single-pulse discharge did not always last until the end of the pulse width, the discharge waveform of each single-pulse discharge was recorded using an oscilloscope (DSO-X2024AS, Agilent Technology Co., Ltd., Santa Clara, CA, USA) in order to calculate the actual discharge duration and discharge energy. The discharge waveforms during a normal discharge are as shown in Figure 3, in which the output voltage (blue line), interelectrode voltage (red line), and current-limiting resistance voltage (orange line) were monitored and recorded. 

### 2.2. Experimental Procedure

First, the workpiece was prepared by cutting the copper sheet with a thickness of 300 μm into 32 mm × 50 mm pieces. Then, the specimens were polished into mirrors to facilitate the observation of a discharge crater. Because the diameter and depth of the discharge craters are generally in the micron or sub-micron level, it is almost impossible to distinguish discharge craters on rough surfaces. Therefore, it is necessary to prepare the sample by polishing it. 

Second, the discharge experiment was carried out. During the discharge experiment, the interelectrode voltage and current-limiting resistance voltage were monitored using the oscilloscope. Before each discharge, a voltage of 15 V was applied to the workpiece and tool, and the tool was fed slowly to the workpiece step by step until the voltage between the workpiece and tool became zero, which was when the tool was in contact with the workpiece. Then, the gap between the workpiece and tool electrode was adjusted to 10 μm, and the workpiece and tool were connected to a power supply system, which was set to the intended open-circuit voltage and pulse width. The tool was fed to the workpiece step by step at a rate of 0.3 µm/step, and a single pulse was emitted after each step until discharge occurred. The tool was then made to return to its original position and was prepared for the next single-pulse discharge experiment. 

Finally, statistical analysis of the experimental results were undertaken. After the discharge experiment, the workpiece was cleaned using an ultrasonic cleaner to remove debris particles adhering to the surface of the workpiece. Then, the radius and shape of the discharge craters were observed and measured using an optical microscopy system (MX-6R, EAST IMAGE, Shanghai, China). 

## 3. Results and Discussion

### 3.1. Effect of the Discharge Duration on the Discharge Energy

The experimental results show that when the pulse width exceeded 10 μs, multiple discharges often occurred during a single pulse period. To avoid errors, this abnormal situation was removed from the statistical data. 

**Figure 4:**
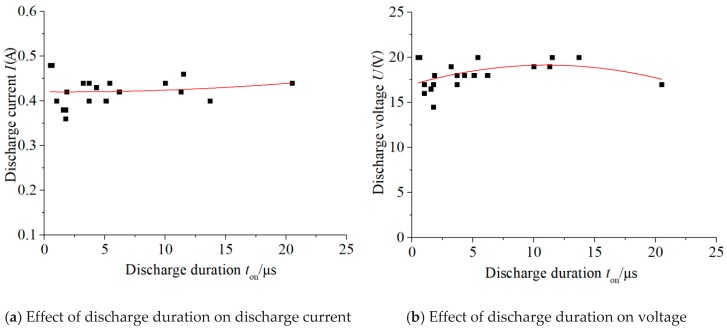
Effect of the discharge duration on the discharge current and voltage.

The effects of the actual discharge duration on the average discharge voltage and average discharge current are shown in Figure 4, in which the discharge current was obtained by dividing the current-limiting resistance voltage by its resistance value. As can be seen from Figure 4, both the discharge voltage and discharge current fluctuated in a certain range, which were immune to the open-circuit voltage and pulse width. 

When the discharge plasma formed after the dielectric breakdown, the circuit changed from being open-circuit to state-of-charge, and the interelectrode resistance became a finite value. Simultaneously, the interelectrode voltage dropped rapidly from an open-circuit voltage to a discharge voltage, and the current of the circuit changed from 0 to a discharge current. After the dielectric breakdown, a discharge plasma channel formed, expanded, and reached a transient state of a shape–position equilibrium. The resistance value of the discharge channel could be obtained by dividing the discharge voltage by the discharge current, which was in the range of 38–45 Ω. During the process of the micro-scale spark discharge, the charged particle movement in the plasma channel was very irregular; therefore, the resistance of the discharge plasma fluctuated continuously. As a result, the constant current-limiting resistance and the fluctuating discharge plasma resistance caused the weak fluctuation of the discharge voltage and discharge current in a certain range, independent of the open-circuit voltage and discharge duration. 

**Figure 5:**
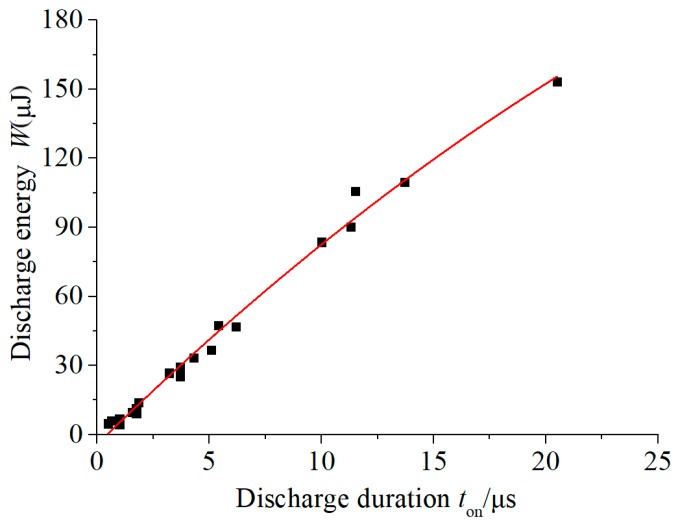
The effect of the discharge duration on the discharge energy.

The variation trend of the discharge energy with the discharge duration is shown in Figure 5, in which the discharge energy was obtained by the product of the discharge voltage, current, and discharge duration. 

It can be observed that the discharge energy was positively correlated with the discharge duration in a standard linear manner. As both the discharge voltage and current can be considered constant, the discharge energy linear increased as the discharge duration increased. It was found that when the pulse width was only a few microseconds, it was easy to generate a single-pulse discharge. When the pulse width exceeded 10 μm, multiple discharges or single discharges that directly turned into a short circuit often occurred during a single-pulse discharge. This was because the micro-scale spark discharge was extremely unstable, and the discharge gap was very small (about 3–5 μm). During the discharge process, the steam torch and debris particles easily caused a short circuit, leading to the deterioration of a discharge state. As a result, because the actual discharge duration was unstable, the discharge energy did not continue to increase with the increase of the pulse width, which made the micro-EDM process more difficult to control. Therefore, micro-EDM was usually accompanied with aided vibration or tool rotation. 

### 3.2. Effect of Actual Discharge Duration on the Radius of a Discharge Crater

**Figure 6:**
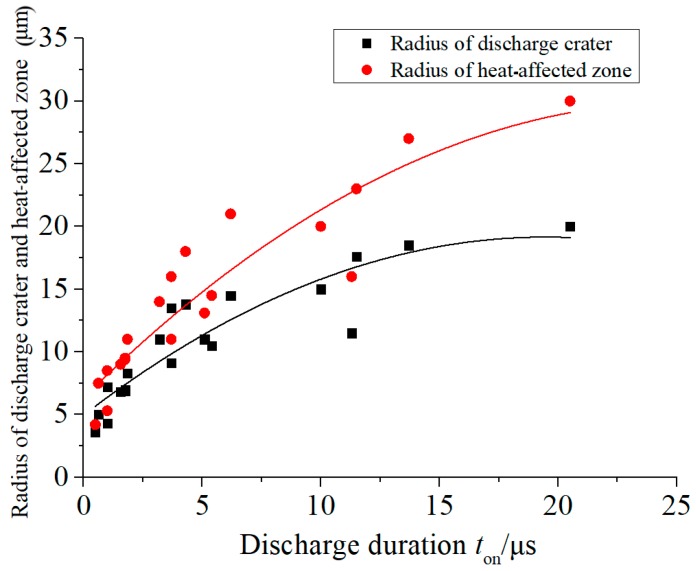
Effect of discharge duration on discharge crater and heat-affected zone radius.

The effects of the discharge duration on the radius of a discharge crater and heat-affected zone are illustrated in Figure 6. When observing and measuring the discharge craters, it was found that there was almost no material removal when the workpiece was connected to a negative polarity. Therefore, only the radius, depth, and related discharge waveform data of the discharge craters obtained when the workpiece was connected to a positive polarity were used for analysis in this study. 

As can be seen from Figure 5, the radius of heat-affected zone and discharge crater increased with the discharge duration, which were consistent in the case of a short-pulse discharge (<4 μs). The discharge channel temperature was quite high when the dielectric broke down, such that the heat did not have enough time to dissipate. Therefore, the discharge energy transferred to the electrode was mainly consumed by the material vaporization and melting. Consequently, in the case of a discharge with a short pulse, the energy utilization rate was relatively high. However, in the case of a long pulse (>15 μs), the radius of the heat-affected zone still increased slowly while the radius of the discharge crater scarcely increased. With the increase of the discharge duration, the discharge channel expanded rapidly and the energy density decreased rapidly, resulting in the increase of molten materials. Simultaneously, the heat-affected zone continued to increase due to the continuous thermal transmission to the surface of workpiece electrodes. As a result, the longer the discharge duration was, the higher the energy efficiency of the micro-EDM. 

Regression models for the discharge crater radius *R* and discharge duration *t* of a single-pulse discharge can be written as follows:

(1) $$R=K\cdot t^{n},$$

where *K* and n are constant.

In order to simplify the process of the regression calculation, natural logarithms were taken on both sides of Equation (1):

(2) lnR=lnK+nlnt.

Thus, the non-linear relationship of the regression model was transformed into a simple linear relationship. The empirical formula of the discharge crater radius *R* of the micro-EDM was obtained by using the statistical software Minitab 15 (Kozo Keikaku Engineering Inc., Tokyo, Japan) for regression analysis, and is given as Equation (3):

(3) $$R=5.7546\cdot t^{0.43}.$$

**Figure 7:**
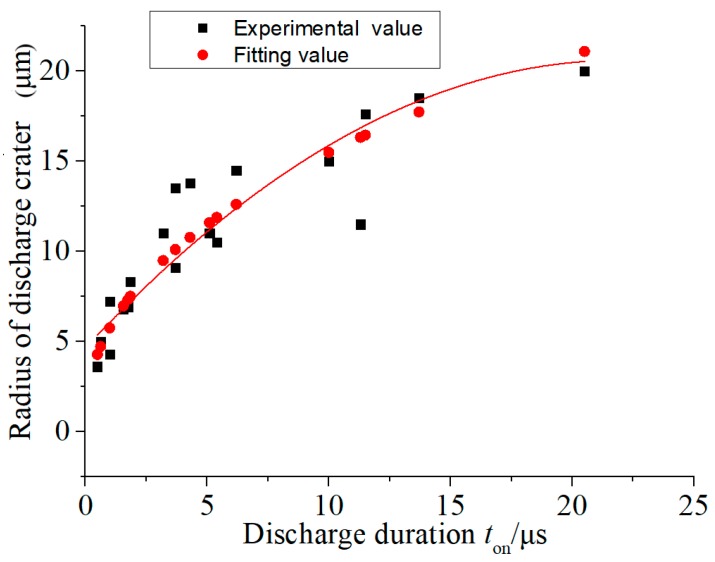
Experimental and fitting values for the discharge crater radius.

The fitting calculation was conducted based on Equation (3). The experimental and fitting values of the discharge crater radius of micro-EDM are shown in Figure 7. It can be seen from the figure that the fitting value was consistent with the experimental value. Although the shape and size of the discharge craters fluctuated within a certain range due to the random characteristics of micro-EDM, the evolution of the discharge crater radius with discharge duration was still clear. Equation (3) fit significantly well, and its R^2^ value was 87.4%. Therefore, the fitting equation played a guiding role in micro-EDM with copper as workpiece. 

### 3.3. Polarity Effect of the Discharge Channel of Micro-EDM

**Figure 8:**
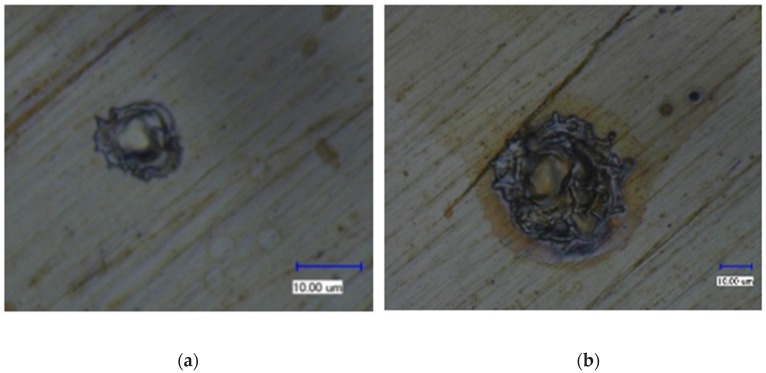
Surface morphology of a discharge crater when the workpiece was connected to a positive electrode: (**a**) the discharge duration was 0.63 μs and (**b**) the discharge duration was 11.5 μs.

**Figure 9:**
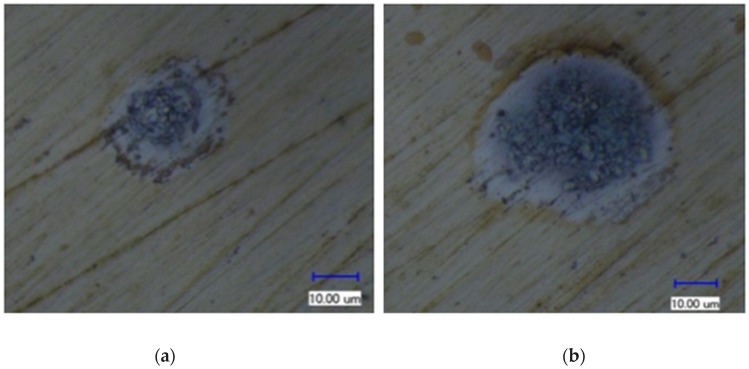
Surface morphology of a discharge crater when the workpiece was connected to a negative electrode: (**a**) the discharge duration was 0.85 μs and (**b**) the discharge duration was 9 μs.

In the single-pulse experiment, it was found that a short circuit was more likely to occur when the workpiece was connected to a negative polarity, and it was difficult to obtain a discharge duration of more than 6 μs. In addition, the shape and size of the discharge craters were quite different when the workpiece connected to a negative polarity compared with those when the workpiece connected to a positive polarity, showing a significant polarity effect. Figure 8 and Figure 9 show the surface morphology of the discharge crater when the workpiece was connected to a positive polarity and a negative electrode, respectively. 

Material removal during the spark discharge was based on the energy conversion from kinetic energy to thermal energy of the charged particles (electrons and ions) in the discharge channel. After a dielectric breakdown, many charged particles moved at high speed in the discharge channel, accompanied by intense particle collisions. The instantaneous high pressure and high temperature made the electrode material melt, vaporize, and eject from the electrode surface. The electrons and positive ions in the discharge channel moved to the positive and negative electrodes, respectively, and their different masses led to different velocities, which resulted in significant differences in the erosion characteristics of the positive and negative electrodes. Since the mass of an electron is far less than that of positive ions, the acceleration of each electron was very large, and the velocity of each electron could be much faster than that of a positive ion in a short time. Therefore, it is generally believed that the energy transferred from an electron to a positive electrode is larger than that transferred from a positive ion to a negative electrode in short-pulse spark discharge. However, the kinetic energy of positive ions is larger than that of an electron when sufficient acceleration time is obtained, such as when the discharge duration is long, resulting in more energy being brought to a negative electrode by positive ion. 

Since the spark discharge is an extremely complex process, it is impossible to calculate the initial position and velocity of charged particle. Assuming that the initial velocity of charged particle is 0, it moves from one electrode surface to another without collision under the effect of an electric field. In this way, the electric field force *F* on charged particles can be expressed as:

(4) $$F=q_{0}\frac{U}{l_{g}},$$

where *F* is the electric field force (N), and $q_{0}$ is the charge of the particle (C), *U* is the discharge voltage (V), and *l*_g_ is the discharge gap (m).

The acceleration of particle can be obtained from Newton’s second law:

(5) $$a=\frac{F}{m_{p}}$$

where *a* is the acceleration of particle (m/s^2^), and $m_{p}$ is the mass of charged particle (kg).

The time and velocity of the motion of the charged particle can be expressed by:

(6) $$\{\begin{array}{c} \frac{1}{2}at^{2}=l_{g},\\ v=at. \end{array}$$

By substituting Equations (4) and (5) into Equation (6), the expressions of motion for time and velocity of charged particles can be written as:

(7) $$\{\begin{array}{c} t=\sqrt{\frac{2m_{p}l_{g}^{2}}{qU}},\\ v=\sqrt{\frac{2qU}{m_{p}}}. \end{array}$$

The known electron charge is 1.6021892 × 10^−19^ C and the mass is 9.10938215 × 10^−31^ kg. It is assumed that the positive ion charge is 1.6021892 × 10^−19^ C and the mass is 3.5 × 10^−25^ kg. By substituting the experimental data into Equation (6), the movement time of an electron and positive ion were calculated to be 3.18 × 10^−12^ s and 1.97 × 10^−9^ s, respectively. The final velocities of an electron and positive ion were 2.52 × 10^6^ m/s and 4.02 × 10^3^ m/s, while the final kinetic energy of an electron and positive ion was 2.89 × 10^−18^ J and 8.17 × 10^−19^ J, respectively. 

From the above calculation, it can be found that the moving time of a charged particle in the discharge channel was much shorter than the discharge duration. Because the discharge gap was very small and there was not enough time for each positive ion to accelerate enough, the final velocity of each electron was three orders of magnitude higher than that of each positive ion. Even if the positive ion had a much larger mass than electron, the kinetic energy of each positive ion was still less than that of each electron when bombarding the surface of the electrode. Moreover, the acceleration and speed of each electron were very high, and the number of times that electrons bombarded the surface of the positive electrode was more than 600 times that of positive ions bombarding the surface of the negative electrode in the same time. In this way, the surface of the positive electrode was bombarded by an avalanche of high-speed electrons, while the surface of the negative electrode was bombarded by positive ions sporadically, resulting in significant differences in the shape and size of the discharge craters. The energy density at the contact point between the discharge channel and the positive electrode surface was much greater than that at the contact point with the negative electrode surface. In order to obtain a higher material removal rate and lower tool wear rate, the workpiece should be connected to the positive polarity in micro-EDM.

## 4. Conclusions

In this study, the variation trends of discharge energy, radius, depth, and volume of discharge crater with discharge duration were discussed based on the experimental results of a single-pulse discharge of micro-EDM. The polarity effect of micro-EDM was analyzed according to the motion characteristics of electron and positive ions in the discharge plasma channel. The main conclusions are as follows:

(1) The discharge maintenance voltage and current were not affected by the discharge duration and open-circuit voltage. The discharge state of micro-EDM was unstable, and different discharge durations were produced by the same pulse width, which was an important reason for the randomness of discharge characteristics.
(2) The energy efficiency of a short-pulse discharge was relatively high, which decreased gradually with the increase of discharge duration.
(3) The movement time of charged particles in the discharge channel was much shorter than the discharge duration due to the very small discharge gap.
(4) The final velocity of each electron was three orders of magnitude higher than that of each positive ion. Even if each positive ion has a much larger mass than an electron, the kinetic energy of the positive ion was still less than that of the electron when bombarding the electrode surface.
(5) Micro-EDM showed a very significant polarity effect. In the same time, the number of times that electrons bombarded the surface of the positive electrode was more than 600 times that of the positive ions bombarding the surface of the negative electrode, therefore there was more material being removed from the positive electrode than the negative electrode.
